# Supplementary material for: The causal relationship between the human gut microbiota and pyogenic arthritis: a Mendelian randomization study
Source: Front Cell Infect Microbiol. 2024 Nov 26;14:1452480. doi: 10.3389/fcimb.2024.1452480 (PMC11629706; doi:10.3389/fcimb.2024.1452480)
Supplement: Supplementary file 1 [file DataSheet1.zip › mendelian test/ebi-a-GCST90027716.csv_forest.pdf]

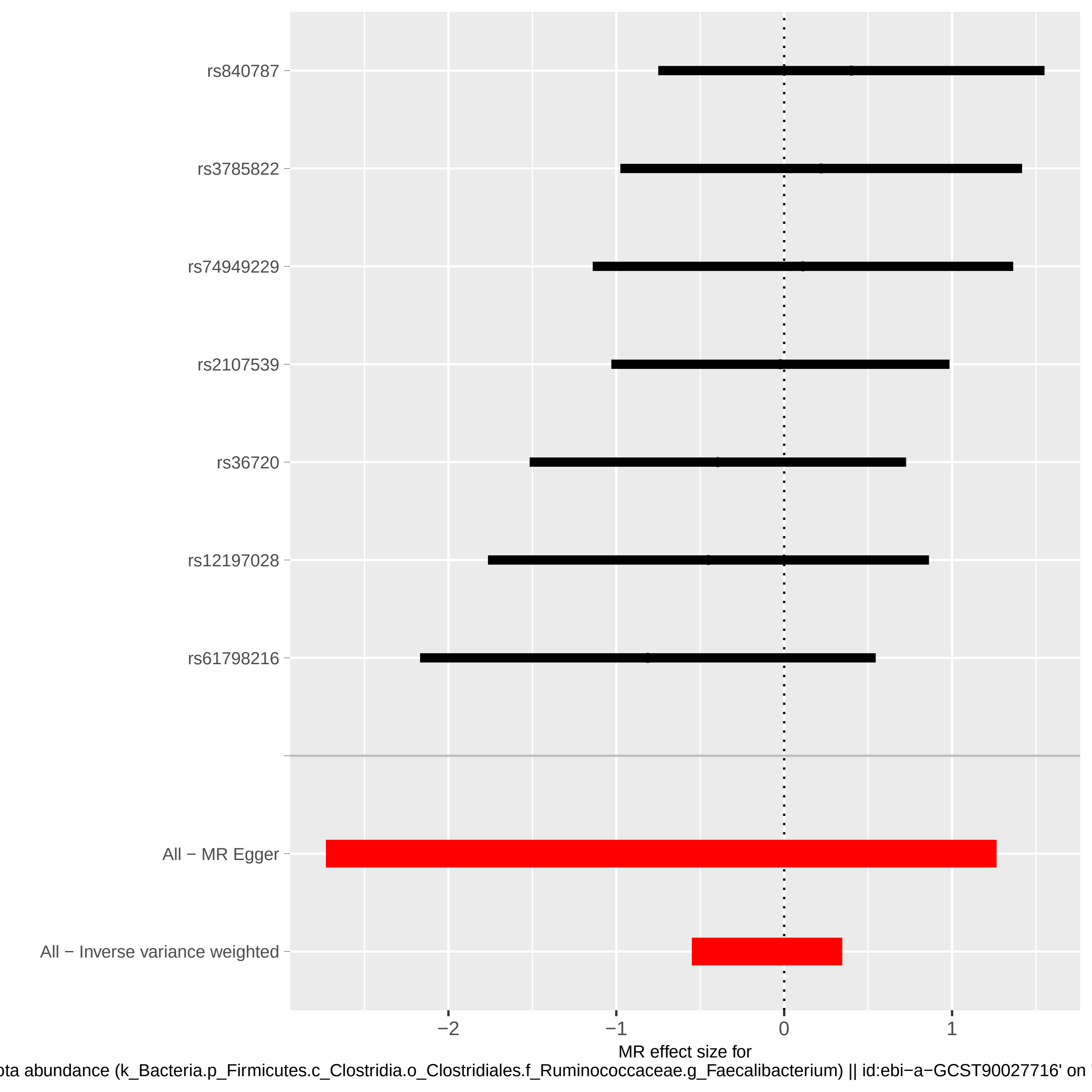
rs840787
rs3785822
rs74949229
rs2107539
rs36720
rs12197028
rs61798216
All − MR Egger
All − Inverse variance weighted
−2
−1
0
1
MR effect size for
ota abundance (k_Bacteria.p_Firmicutes.c_Clostridia.o_Clostridiales.f_Ruminococcaceae.g_Faecalibacterium) || id:ebi−a−GCST90027716' on
